# Causal association between atopic dermatitis and Parkinson's disease: A bidirectional Mendelian randomization study

**DOI:** 10.1002/brb3.3468

**Published:** 2024-03-11

**Authors:** Taofeng Zhou, Baohao Wei, Yachun Hu, Xiaoming Zhou, Xiaoying Cai, Xiaolei Shi

**Affiliations:** ^1^ Department of Neurology Yijishan Hospital, Wannan Medical College Wuhu China; ^2^ Department of Neurology The Affiliated Brain Hospital of Guangzhou Medical University Guangzhou China; ^3^ Department of Anesthesiology The First Affiliated Hospital of Sun Yat‐Sen University Guangzhou China; ^4^ Geriatric Neuroscience Center The Affiliated Brain Hospital of Guangzhou Medical University Guangzhou China; ^5^ Guangdong Engineering Technology Research Center for Translational Medicine of Mental Disorders Guangzhou China; ^6^ Key Laboratory of Neurogenetics and Channelopathies of Guangdong Province and the Ministry of Education of China Guangzhou Medical University Guangzhou China

**Keywords:** atopic dermatitis, causal association, Mendelian randomization, Parkinson's disease

## Abstract

**Background:**

Atopic dermatitis is one of the most common skin disorders. Evidence has suggested an association between skin disorders, such as atopic dermatitis, and Parkinson's disease (PD). However, whether atopic dermatitis has a causal effect on PD remains unknown.

**Methods:**

The study aimed to determine whether their association between atopic dermatitis and PD is causal, using a bidirectional two‐sample Mendelian randomization method. Genetic variants from the public genome‐wide association studies for atopic dermatitis (*n* = 10788 cases and 30047 controls) were selected to evaluate their causal effects on the risk of PD (33,674 cases and 449,056 controls). The inverse variance weighted (IVW) method was used as the primary analysis.

**Results:**

The IVW results indicated that atopic dermatitis was associated with decreased risk of PD {fixed effects: odds ratio [OR] [95% confidence interval (CI)]: .905 [.832–.986], *p* = .022; OR [95% CI]: .905 [.827–.991], *p* = .032}. However, we failed to detect the causal effects of PD on risk of atopic dermatitis in the reverse causation analysis.

**Conclusion:**

This study indicated causal association of genetically proxied atopic dermatitis with the risk of PD. Future studies are warranted to explore the underlying mechanism and investigate the targeting effect of atopic dermatitis on PD.

## Graphical abstract

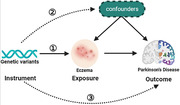

## 1 INTRODUCTION

Parkinson's disease (PD) is a neurodegenerative disorder, which is characterized by progressive degeneration of dopamine neurons in brain (Jankovic, 2008; Vernier et al., 2004), and it affects a great number of people around the world. In recent years, the skin presentations of PD are attracting increasing attentions. For example, seborrheic face is recognized as a featured manifestation of PD (Niemann et al., 2021). Besides, Tomic et al. (2022) reported that seborrheic dermatitis was associated with the severity of motor symptoms of PD. Therefore, it is important to investigate the relationship between PD and skin disorders, which helps better understanding of PD.

Atopic dermatitis is the most common chronic, inflammatory, relapsing skin disorder, with a prevalence of 7%–14% among adult population (Bylund et al., 2020). It mainly presents with symptoms, including severe itching and recurring eczematous lesions (Li et al., 2021), and imposes a great burden to patients and their family (Drucker, 2017). Emerging evidence has suggested that atopic dermatitis is associated with depression and anxiety (Baurecht et al., 2021), which are common symptoms of PD (Pontone & Mills, 2021), pointing out the potential link between atopic dermatitis and PD.

Their association is likely to involve the inflammatory nature of the two disorders. Many studies have indicated the association of atopic dermatitis with an immune response of the cell community through the human body, including Type 1 T helper (Th1), Type 2 T helper (Th2), and Regulatory T (Treg) cells (Auriemma et al., 2013). It is believed that an alteration of Th1‐ and Th2‐mediated immune responses and IgE‐regulated hypersensitivity reactions is responsible for the development and progression of the disorder. For PD, systemic inflammation helps explain the onset, development, and progression of the disorder (Tansey & Romero‐Ramos, 2019). Thus, atopic dermatitis might have a distinct role in the pathology of PD. However, a cohort study by Nam et al. (2024) did not reveal a significant increased risk of PD in patients with atopic dermatitis. Then, it is crucial to investigate whether there is a causal relationship between atopic dermatitis and PD remains unclear.

Mendelian randomization (MR) analysis provides a method to estimate the causal effect of a certain risk factor on an outcome of interest (such as a disease), using genetic variants as instrumental variables (IVs) (Skrivankova et al., 2021). MR has been increasingly applied in epidemiology and clinical studies, for its less susceptibility to unmeasured confounding factors and potential reverse causation (Burgess et al., 2021). In this study, we used the MR method to explore the causality between atopic dermatitis and risk of PD.

## 2 MATERIALS AND METHODS

### 2.1 Data sources

Publicly available data were used in this study, including genome‐wide association studies (GWAS) data for atopic dermatitis and PD. Informed patients consents and ethical approvals were available in each data center. Summary genetic association estimates for PD were extracted from the International PD Genomics Consortium (IPDGC) (Nalls et al., 2019), including 33,674 cases and 449,056 controls of European ancestry. Summary level statistics for atopic dermatitis were from the study by Paternoster et al. (2015), including 10,788 cases and 30,047 controls from 20 studies of European ancestry, excluding 23andMe study.

### 2.2 Selection of SNPs

**FIGURE 1:**
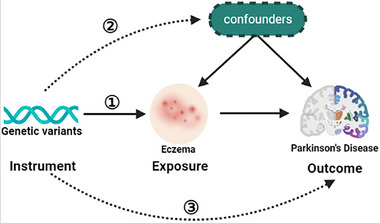
Graphic representation of the Mendelian randomization (MR) design. Three principal assumptions should be fulfilled in the MR study: (1) genetic instrumental variants should be stably associated with eczema; (2) genetic instrumental variants should not be associated with any confounding factors; and (3) genetic instrumental variants should only influence the risk of Parkinson's disease (PD).

Under the design of MR (Figure 1), single nucleotide polymorphisms (SNPs) for exposure were selected as IVs when they met three key assumptions. Based on these principals, we did the following processes to select suitable IVs. First, SNPs should passed the genome‐wide significance criteria (*p* < 5e − 08). Then they were pruned based on the linkage disequilibrium (*r* ^2^ = .001, clumping distance = 10,000 kb) to ensure the independence of each SNP. Moreover, we calculated the *F* statistic to ensure the strength of the exposures, and *F* statistic > 10 indicated that the screening IVs were strongly correlated with the exposure. The *R* ^2^ and *F* statistics of each SNP were calculated based on the formulas: *R* ^2^ = 2 × EAF × (1 − EAF) × *β* ^2^ and *F* statistics = *R* ^2^ × (*N *− 2)/(1 − *R* ^2^). After that, the instrument SNPs were extracted from the outcome GWAS dataset. Finally, variants and their alleles from the exposure and outcome data were harmonized to ensure that the beta coefficients and standard errors for the exposure and outcome correspond to the same allele.

### 2.3 MR analysis

We carried out two‐sample MR analysis to explore the causal effects of eczema on risk of PD, using the “TwoSampleMR” package of R software. Both fixed‐ and random‐effects inverse variance weighted (IVW) models were conducted as the primary analysis. The IVW model assumes that all SNPs included for the estimation of causal effects are valid (Mounier & Kutalik, 2023). Alternative MR methods were conducted for supplements, including simple median, MR‐Egger, and maximum likelihood methods (Bowden et al., 2017; Burgess & Thompson, 2017; Hemani et al., 2018). Cochran's *Q* test in IVW model was performed to detect the heterogeneity among SNPs in the respective analysis. The MR‐Egger regression intercept was applied to detect the presence of horizontal pleiotropy, and a *p*_intercept_ less than .05 was considered significant for the presence of horizontal pleiotropy. Furthermore, we used the MR pleiotropy residual sum and outlier (MR‐PRESSO) global test to detect and correct for horizontal pleiotropic outliers (Verbanck et al., 2018). Leave‐one‐out test was done to detect the effect of one single SNP on the outcome.

## 3 RESULTS

### 3.1 Genetic variants as instrumental variables

A total of 12 SNPs were identified as IVs for atopic dermatitis. The *F* statistics of them were all above 10, ranging from 174.062 to 419.088. The detailed information of SNPs associated with atopic dermatitis and PD are shown in Tables S1 and S2, respectively.

### 3.2 Two‐sample MR of atopic dermatitis and PD

**FIGURE 2:**
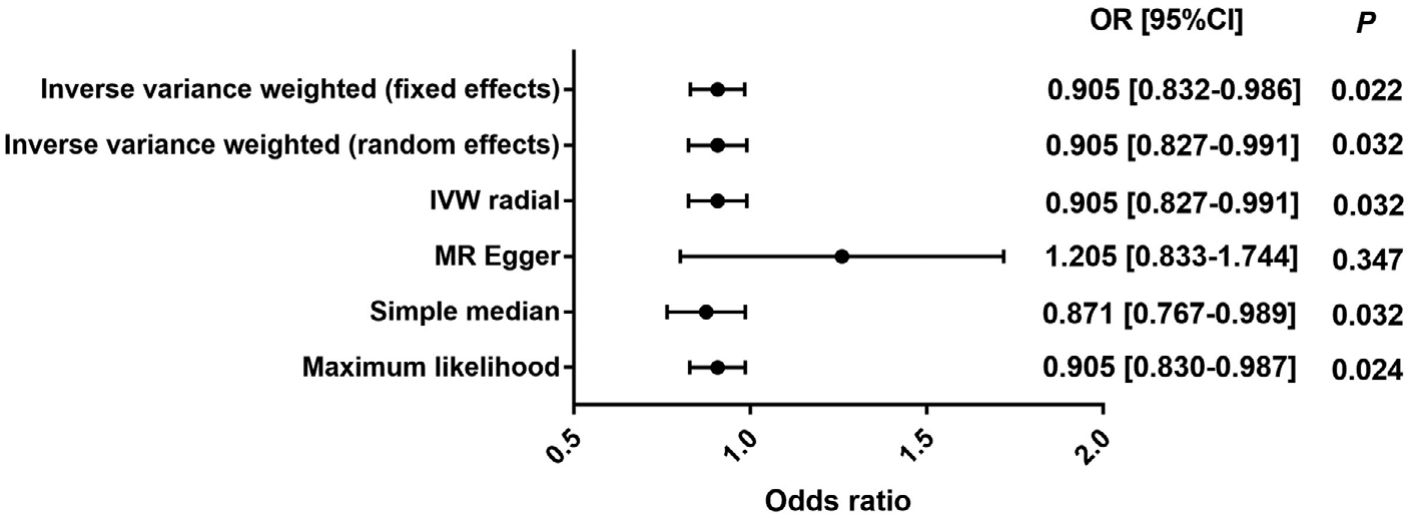
Mendelian randomization (MR) results of the association between atopic dermatitis and Parkinson's disease (PD). CI, confidence interval; OR, odds ratio.

**FIGURE 3:**
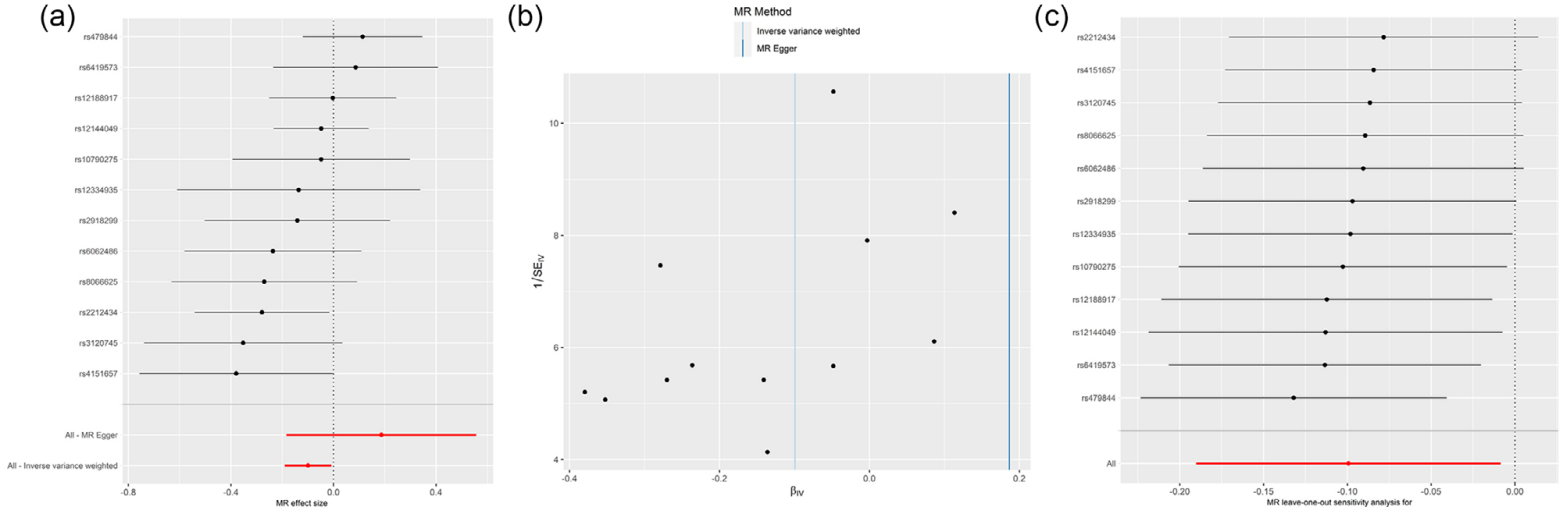
Forest plot (A), scatter plot (B), and leave‐one‐out analysis (C) of the causal effect of atopic dermatitis on Parkinson's disease (PD).

The complete sets of MR results are displayed in Table 1 and Figure 2. The results of both fixed‐ and random‐effect IVW analyses showed a causal effect of atopic dermatitis on risk of PD (odds ratio [OR] = .905, 95% confidence interval [CI]: .832–.986, *p *= .022; OR = .905, .827–.991, *p *= .032). Similar results were observed in the models of simple median, maximum likelihood, and IVW radial. Unfortunately, the MR‐Egger regression model results did not show statistically significance. No heterogeneity was detected by Cochran's *Q* test among the IVs (*Q* = 12.541,*p* = .324) (Table 2). Moreover, no horizontal pleiotropy was detected using the MR‐Egger intercept (*p*_intercept_ = .150) (Table 2). The MR‐PRESSO analysis did not detect any instrumental outlier at the significance level of .05 (Table 2). A leave‐one‐out analysis was done to test the conformity of the included SNPs, and it indicated that no specific SNP significantly affected the overall MR results. The forest plot, funnel plot, and leave‐one‐out analysis results are presented in Figure 3.

**TABLE 1:** Mendelian randomization (MR) results of the association between atopic dermatitis and Parkinson's disease (PD).

Exposure	Outcome	Model	No. of SNPs	*β*	SE	OR [95% CI]	*p* Value
Eczema	PD	IVW (fixed effects)	12	−.099	.043	.905 [.832–.986]	.022
		IVW (random effects)	12	−.099	.046	.905 [.827–.991]	.032
		IVW radial	12	−.099	.046	.905 [.827–.991]	.032
		MR Egger	12	.187	.189	1.205 [.833–1.744]	.347
		Simple median	12	−.139	.065	.871 [.767–.989]	.032
		Maximum likelihood	12	−.100	.044	.905 [.830–.987]	.024

Abbreviations: CI, confidence interval; IVW, inverse variance weighted; OR, odds ratio; SE, standard error; SNP, single nucleotide polymorphisms.

**TABLE 2:** Heterogeneity and horizontal pleiotropy analysis between atopic dermatitis and Parkinson's disease (PD).

Heterogeneity			Horizontal pleiotropy			MR‐PRESSO
*Q*	df`	*p* Value	Egger intercept	SE	*p Value*	*p* Value
12.541	11	.324	−.040	.026	.150	.354

Abbreviation: SE, standard error.

**FIGURE 4:**
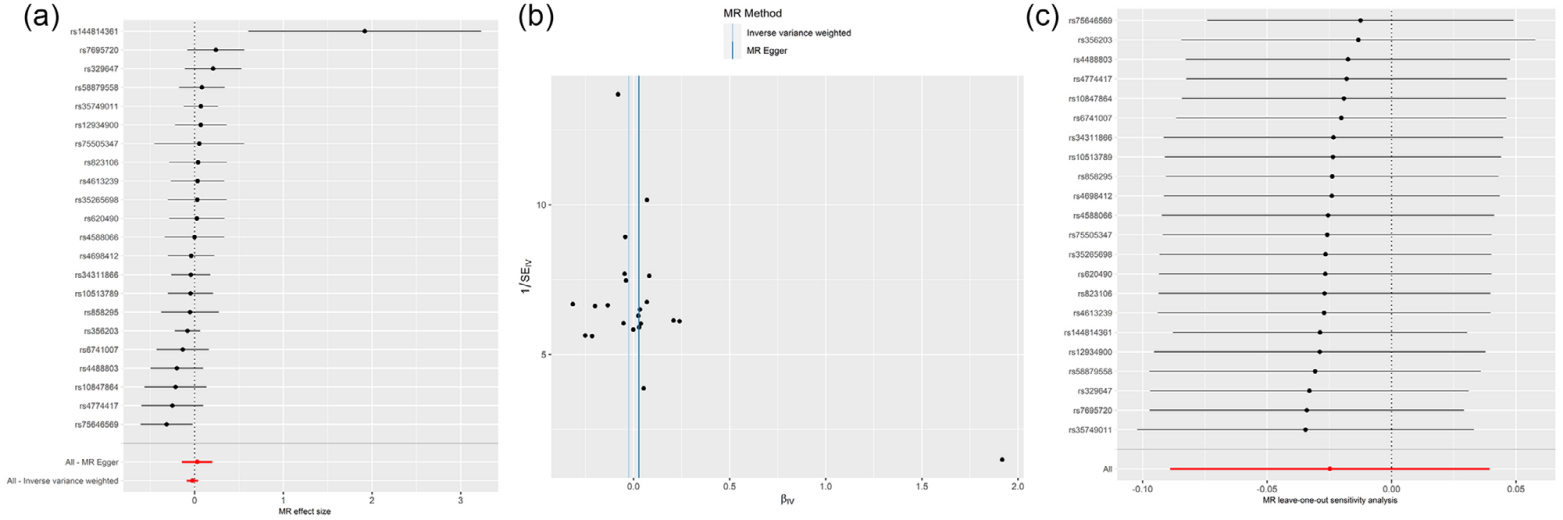
Forest plot (A), scatter plot (B), and leave‐one‐out analysis (C) of the causal effect of Parkinson's disease (PD) on atopic dermatitis.

Reverse MR analysis of the effects of PD on risk of atopic dermatitis was done and indicated no causality between the two disorders (Table S3). The heterogeneity and horizontal pleiotropy analysis are shown in Table S4. The scatter plot, forest plot, funnel plot, and leave‐one‐out analysis results are presented in Figure 4.

## 4 DISCUSSION

In the current study, we leveraged the effects of atopic dermatitis on risk of PD by identifying genetic variants as IVs for the analysis of their associations. This MR study indicated that atopic dermatitis was negatively associated with risk of PD. No evidence in the reverse MR analysis was observed. These provided a better understanding of the role of atopic dermatitis in PD.

Dermatological diseases, such as seborrheic dermatitis, pemphigoid, and melanoma, are recognized as common non‐motor symptoms of PD (Niemann et al., 2021). Cumulative studies have indicated an increased prevalence of skin disorders among patients with PD (Niemann et al., 2021), even before the presentation of motor symptoms (Pont‐Sunyer et al., 2015). In a previous study by Tanner et al. (2012), the authors demonstrated that seborrheic dermatitis might represent a premotor feature of PD, ascribable to the dysregulation of autonomic nervous system, and suggested that this skin disorder could serve as an early disease indicator. The explanation for the association between dermatological disorders and PD remains unclear, but various hypotheses have been proposed. Among them, a potential genetic link was raised by researchers. For instance, the association of seborrheic dermatitis with PD may originate from the shared genetic polymorphisms of GBA, LRRK2, and PINK1 (Trinh & Farrer, 2013), which play a role in immune changes, lipid metabolism, and homeostasis of brain (Dzamko et al., 2015; Magnusen et al., 2021). Then, it is important to investigate the underlying genetic link between skin diseases and PD.

MR study helps understand the causality between certain exposures and outcomes, by applying genetic variants as IVs (Skrivankova et al., 2021). The causality between skin disorder and PD has also been established in a previous MR study focusing on psoriasis (Li et al., 2022), which indicated that higher psoriasis risk was nominally associated with progression of PD. The associations may originate from the immune activities in psoriasis (Afonina et al., 2021). Psoriasis is a common skin disease, which represents marked inflammatory changes, including keratinocyte hyperproliferation and altered differentiation and infiltration of leukocytes in skin tissues (Eberle et al., 2016; Ortiz‐Lopez et al., 2022). Patients with psoriasis are likely to represent chronic periphery inflammation (Reich, 2012), which could have an essential role in the development of PD (Williams et al., 2022). It is believed that systemic inflammation is responsible the activation of microglia, the production of reactive oxygen species, and neuronal damages (Hoogland et al., 2015). These changes are of particular importance for the degeneration of substantia nigra, and other parts in PD brains (Marinova‐Mutafchieva et al., 2009). Moreover, the inflammatory components could increase the accumulation of α‐synuclein, which in turn causes activation of immune cells and the production of inflammatory factors (Allen Reish & Standaert, 2015).

Interestingly, our study indicated that atopic dermatitis is negatively associated with risk of PD through an MR framework. Results were confirmed in both fixed‐ and random‐effects IVW models, as well as most MR models. This was not consistent with the associative trend between psoriasis, which also shares an immune cause with atopic dermatitis and PD. Atopic dermatitis is a chronic inflammatory skin disease with clinical manifestations, including intense pruritus and eczematous lesions (Tamagawa‐Mineoka & Katoh, 2020). Intensive immune activities can also be found in atopic dermatitis (Novak, 2003). Studies have indicated that atopic dermatitis is induced by the immune response in human body, including Th1, Th2, and Treg cells, as well as the related immune alterations (Auriemma et al., 2013). Both atopic dermatitis and psoriasis are common T‐cell mediated inflammatory diseases of the skin, and they can be treated by specific cytokine antagonists or immunosuppressive agents. In addition, they are quite similar in the alterations of epidermal keratinocytes when responding to T‐cell produced cytokines. However, the two skin disorders display varied T‐cell polarity and different spectrums of cytokines (Guttman‐Yassky & Krueger, 2017). Specially, psoriasis is driven by a single polar immune pathway mainly associated with Th17 T‐cells and activation of IL‐17, whereas atopic dermatitis is driven by multiple polar immune pathways. For instance, Th2 and Th22 are two major T‐cell subsets commonly present across the subtypes of atopic dermatitis. Therefore, the opposite causal trends of atopic dermatitis and psoriasis with PD may be originated from the participation of different T‐cell subsets, and other unknown immune components.

The main advantage of the study is that a bidirectional MR design was applied to avoid reverse causality and confounding factors as much as possible. In addition, no evidence is available of the reverse association analysis between eczema and PD, ensuring the stability of the results. More studies should be done to explore the potential mechanisms.

### 4.1 Limitation

However, our study also has limitations. First, MR study might be influenced by the ethnic population sources. A recent study by Nam et al. (2024) analyzed the associations of allergic diseases, such as allergic rhinitis, asthma, and atopic dermatitis with PD, and they did not find a significant increase in the risk of PD among patients with atopic dermatitis. The participants in that study were included from the Korean NHIS database, which covered approximately 98% of the population in South Korean. This is quite different from our study that the summary‐level GWAS datasets used in our study were mainly from people of European ancestry. Therefore, our findings should be applied with caution when generalized to other ancestry groups. Second, a major limitation for MR analysis is the potential pleiotropic effects. Further studies with larger sample size should help solve it. Third, MR‐Egger model referred to a nonsignificant relationship between atopic dermatitis and PD. Although MR‐Egger is desirable as sensitivity analysis as it allows all genetic variants to violate the instrumental variable assumptions to assess the robustness of statistical results, it requires all genetic variants to satisfy an alternative assumption (Burgess & Thompson, 2017). Therefore, MR‐Egger is far from the only solution for sensitivity analysis in MR.

## 5 CONCLUSION

The current project identified genetic variants to instrument the effects of eczema and employed them in the MR framework to generate genetic evidence supporting the causal effects of decreased incidence of eczema on risk of PD.

## AUTHOR CONTRIBUTIONS

*Conceptualization; formal analysis; writing—original draft; writing—review and editing; supervision*: Taofeng Zhou. *Methodology; validation; formal analysis; writing—original draft*: Baohao Wei. *Methodology; data curation*: Yachun Hu. *Data curation; validation*: Xiaoming Zhou. *Methodology; validation*: Xiaoying Cai. *Writing—review and editing; writing—original draft; conceptualization; supervision; funding acquisition*: Xiaolei Shi.

## CONFLICT OF INTEREST STATEMENT

The authors have no conflicts of interest.

### PEER REVIEW

The peer review history for this article is available at https://publons.com/publon/10.1002/brb3.3468.

## Supporting information


**Table S1** The SNPs associated with atopic dermatitis. Chr, chromosome; EAF, effect allele frequency; SE, standard error; SNP, single nucleotide polymorphism.
**Table S2** The SNPs associated with PD. Chr, chromosome; EAF, effect allele frequency; PD, Parkinson's disease; SE, standard error; SNP, single nucleotide polymorphism.
**Table S3** The reverse causation analysis of the effects of PD on atopic dermatitis. CI, confidence interval; IVW, inverse variance weighted; MR, Mendelian randomization; OR, odds ratio; PD, Parkinson's disease; SE, standard error.
**Table S4** Heterogeneity and horizontal pleiotropy analysis between PD and atopic dermatitis. PD, Parkinson's disease; SE, standard error.

## Data Availability

Data used in the study were from the IEU GWAS database (https://gwas.mrcieu.ac.uk/).
