# Supplementary material for: Causal association between atopic dermatitis and Parkinson's disease: A bidirectional Mendelian randomization study
Source: Brain Behav. 2024 Mar 11;14(3):e3468. doi: 10.1002/brb3.3468 (PMC10928334; doi:10.1002/brb3.3468)
Supplement: Supplementary file 1 — Table S1 The SNPs associated with atopic dermatitis. Chr, chromosome; EAF, effect allele frequency; SE, standard error; SNP, single nucleotide polymorphism. Table S2 The SNPs associated with PD. Chr, chromosome; EAF, effect allele frequency; PD, Parkinson's disease; SE, standard error; SNP, single nucleotide polymorphism. Table S3 The reverse causation analysis of the effects of PD on atopic dermatitis. CI, confidence interval; IVW, inverse variance weighted; MR, Mendelian randomization; OR, odds ratio; PD, Parkinson's disease; SE, standard error. Table S4 Heterogeneity and horizontal pleiotropy analysis between PD and atopic dermatitis. PD, Parkinson's disease; SE, standard error. [file BRB3-14-e3468-s001.docx]

| **SNP** | **Chr** | **Position** | **Effect allele** | **Other allele** | **Beta** | **SE** | **EAF** | **Sample size** | **R^2^** | **F** |
| --- | --- | --- | --- | --- | --- | --- | --- | --- | --- | --- |
| rs3120745 | 1 | 1.53E+08 | G | A | -0.10699 | 0.019576 | 0.690562 | 40530 | 0.004892 | 199.2293 |
| rs12144049 | 1 | 1.52E+08 | T | C | -0.20181 | 0.018641 | 0.677644 | 40530 | 0.017792 | 734.147 |
| rs6419573 | 2 | 1.03E+08 | C | T | -0.12395 | 0.01965 | 0.708531 | 40529 | 0.006346 | 258.8114 |
| rs12188917 | 5 | 1.32E+08 | C | T | 0.170064 | 0.021524 | 0.205033 | 40530 | 0.009428 | 385.7419 |
| rs4151657 | 6 | 31917540 | C | T | 0.10198 | 0.017657 | 0.33741 | 40531 | 0.00465 | 189.3446 |
| rs12334935 | 8 | 1.27E+08 | A | G | 0.092611 | 0.016878 | 0.473769 | 40529 | 0.004277 | 174.0624 |
| rs2212434 | 11 | 76281593 | T | C | 0.129133 | 0.016879 | 0.450704 | 40530 | 0.008257 | 337.4102 |
| rs479844 | 11 | 65551957 | G | A | 0.143746 | 0.017038 | 0.548407 | 40531 | 0.010235 | 419.0881 |
| rs10790275 | 11 | 1.19E+08 | C | G | 0.122434 | 0.021853 | 0.7512 | 40834 | 0.005603 | 230.0811 |
| rs8066625 | 17 | 40390629 | A | G | 0.175585 | 0.031912 | 0.107212 | 40529 | 0.005902 | 240.609 |
| rs2918299 | 19 | 8787273 | T | C | 0.142564 | 0.022955 | 0.166014 | 38477 | 0.005628 | 217.7624 |
| rs6062486 | 20 | 62302539 | A | G | 0.104574 | 0.018726 | 0.647639 | 40531 | 0.004991 | 203.2999 |

**Supplemental Table 1.** The SNPs associated with atopic dermatitis. Chr, chromosome; EAF, effect allele frequency; SE, standard error; SNP, single nucleotide polymorphism.

| **SNP** | **Chr** | **Position** | **Effect allele** | **Other allele** | **Beta** | **SE** | **EAF** | **Sample size** | **R^2^** | **F** |
| --- | --- | --- | --- | --- | --- | --- | --- | --- | --- | --- |
| rs35749011 | 1 | 1.55E+08 | A | G | 0.7508 | 0.0659 | 0.0191 | 482730 | 0.021122 | 10416.23 |
| rs823106 | 1 | 2.06E+08 | C | G | -0.1492 | 0.0239 | 0.8488 | 482730 | 0.005714 | 2774.06 |
| rs4613239 | 2 | 1.69E+08 | G | C | 0.1784 | 0.0248 | 0.1326 | 482730 | 0.007321 | 3560.216 |
| rs6741007 | 2 | 1.36E+08 | G | T | -0.1233 | 0.0175 | 0.4507 | 482730 | 0.007528 | 3661.317 |
| rs4488803 | 3 | 58218352 | A | G | -0.1136 | 0.0199 | 0.3746 | 482730 | 0.006047 | 2936.627 |
| rs10513789 | 3 | 1.83E+08 | G | T | -0.1596 | 0.0219 | 0.1826 | 482730 | 0.007604 | 3698.695 |
| rs7695720 | 4 | 77183300 | C | A | -0.1255 | 0.0208 | 0.2091 | 482730 | 0.005209 | 2527.923 |
| rs34311866 | 4 | 951947 | C | T | 0.2272 | 0.0231 | 0.1958 | 482730 | 0.016256 | 7977.08 |
| rs4698412 | 4 | 15737348 | A | G | 0.1258 | 0.0168 | 0.553 | 482730 | 0.007824 | 3806.604 |
| rs356203 | 4 | 90666041 | T | C | -0.2398 | 0.0178 | 0.6169 | 482730 | 0.02718 | 13487.31 |
| rs75646569 | 5 | 60345424 | G | T | 0.1916 | 0.0266 | 0.1117 | 482730 | 0.007285 | 3542.516 |
| rs35265698 | 6 | 32561334 | G | C | -0.2 | 0.0303 | 0.1547 | 480593 | 0.010461 | 5103.416 |
| rs858295 | 7 | 23245569 | G | A | -0.1039 | 0.0176 | 0.3947 | 482730 | 0.005158 | 2502.922 |
| rs620490 | 8 | 16697579 | G | T | -0.1174 | 0.019 | 0.2762 | 482730 | 0.005511 | 2674.92 |
| rs144814361 | 10 | 1.21E+08 | T | C | 0.4411 | 0.068 | 0.0174 | 482730 | 0.006653 | 3233.194 |
| rs329647 | 11 | 1.34E+08 | C | G | -0.1133 | 0.0178 | 0.6662 | 482730 | 0.005709 | 2771.851 |
| rs75505347 | 12 | 40885549 | T | C | 0.3917 | 0.0674 | 0.0195 | 482730 | 0.005867 | 2848.901 |
| rs10847864 | 12 | 1.23E+08 | T | G | 0.1274 | 0.0179 | 0.3625 | 482730 | 0.007502 | 3648.629 |
| rs4774417 | 15 | 61993702 | A | G | 0.1052 | 0.0192 | 0.7397 | 482730 | 0.004262 | 2066.087 |
| rs12934900 | 16 | 30923602 | T | A | 0.1215 | 0.0184 | 0.6571 | 482730 | 0.006652 | 3232.829 |
| rs58879558 | 17 | 44095467 | C | T | -0.2383 | 0.025 | 0.2229 | 482730 | 0.019673 | 9687.16 |
| rs10451230 | 17 | 16035225 | T | A | -0.096 | 0.0175 | 0.565 | 482730 | 0.00453 | 2196.77 |
| rs4588066 | 18 | 40672964 | A | G | 0.1046 | 0.0178 | 0.326 | 482730 | 0.004808 | 2332.204 |

**Supplemental Table 2.** The SNPs associated with PD. Chr, chromosome; EAF, effect allele frequency; PD, Parkinson’s Disease; SE, standard error; SNP, single nucleotide polymorphism.

| **Exposure** | **Outcome** | **Methods** | **No. of SNPs** | **Beta** | **SE** | **OR [95%CI]** | ***P*** |
| --- | --- | --- | --- | --- | --- | --- | --- |
| PD | Eczema | IVW (fixed effects) | 22 | -0.02478 | 0.030129 | 0.976 [0.920-1.035 | 0.411 |
|  |  | IVW (random effects) | 22 | -0.02478 | 0.032729 | 0.976 [0.915-1.040] | 0.449 |
|  |  | IVW radial | 22 | -0.02478 | 0.032731 | 0.976 [0.915-1.040] | 0.449 |
|  |  | MR Egger | 22 | 0.027895 | 0.087161 | 1.028 [0.867-1.220] | 0.752 |
|  |  | Simple median | 22 | 0.011775 | 0.043364 | 1.012 [0.929-1.102] | 0.786 |
|  |  | Maximum likelihood | 22 | -0.02504 | 0.03034 | 0.975 [0.919-1.035] | 0.409 |

**Supplemental Table 3.** The reverse causation analysis of the effects of PD on atopic dermatitis. CI, confidence interval; IVW, Inverse variance weighted; MR, Mendelian randomization; OR, odds ratio; PD, Parkinson’s Disease; SE, standard error.

| **Heterogeneity** | | | **Horizontal pleiotropy** | | | **MR-PRESSO** |
| --- | --- | --- | --- | --- | --- | --- |
| Q | df | P | Egger intercept | SE | P | P |
| 24.780 | 21 | 0.257 | -0.009 | 0.013 | 0.521 | 0.368 |

**Supplemental Table 4.** Heterogeneity and horizontal pleiotropy analysis between PD and atopic dermatitis. PD, Parkinson’s Disease; SE, standard error.
